# Resist-Free Directed Self-Assembly Chemo-Epitaxy Approach for Line/Space Patterning

**DOI:** 10.3390/nano10122443

**Published:** 2020-12-07

**Authors:** Tommaso Jacopo Giammaria, Ahmed Gharbi, Anne Paquet, Paul Nealey, Raluca Tiron

**Affiliations:** 1 Université Grenoble Alpes, CEA, Leti, F-38000 Grenoble, France; ahmed.gharbi@cea.fr (A.G.); anne.paquet74@gmail.com (A.P.); raluca.tiron@cea.fr (R.T.); 2 Institute for Molecular Engineering, University of Chicago, 5747 South Ellis Avenue, Chicago, IL 60637, USA; nealey@uchicago.edu

**Keywords:** directed self-assembly (DSA), block copolymers (BCPs), chemo-epitaxy, polystyrene-block-polymethylmethacrylate (PS-*b*-PMMA), line/space patterning, line edge roughness (LER), line width roughness (LWR)

## Abstract

This work reports a novel, simple, and resist-free chemo-epitaxy process permitting the directed self-assembly (DSA) of lamella polystyrene-block-polymethylmethacrylate (PS-*b*-PMMA) block copolymers (BCPs) on a 300 mm wafer. 193i lithography is used to manufacture topographical guiding silicon oxide line/space patterns. The critical dimension (CD) of the silicon oxide line obtained can be easily trimmed by means of wet or dry etching: it allows a good control of the CD that permits finely tuning the guideline and the background dimensions. The chemical pattern that permits the DSA of the BCP is formed by a polystyrene (PS) guide and brush layers obtained with the grafting of the neutral layer polystyrene-random-polymethylmethacrylate (PS-*r*-PMMA). Moreover, data regarding the line edge roughness (LER) and line width roughness (LWR) are discussed with reference to the literature and to the stringent requirements of semiconductor technology.

## 1. Introduction

The block copolymers (BCPs) have attracted more and more attention because under suitable conditions, they self-assemble in highly ordered polymeric templates with well-defined sub-20 nm periodic features that could be extremely useful for a wide range of nanotechnology applications [1,2,3,4]. The morphology of the self-assembled nanostructures (spherical, cylindrical, gyroid, or lamellar) is determined by the fraction of one of the blocks with respect to the other one that form the BCP chain [5]. The directed self-assembly (DSA) of block copolymers (BCPs) permits obtaining well-ordered nanostructures having high resolution [6]. In this context, the chemo-epitaxial process represents one of the most used approaches to induce the long-range ordering of the BCPs nanostructures by means of the chemical contrast between the guideline and background. In this case, it is possible to obtain a long-range ordering when the dimension of the guideline and background are commensurate with the intrinsic period of the BCP [7,8,9,10]. Numerous methods were developed for the DSA of lamellae forming BCPs [11,12,13,14], but in the framework of 300 mm fab process manufacturing, where all the fabrication processes are made on a 300 mm wafer, LiNe [15], SMART [16], and COOL [17] processes are the most attractive.

The LiNe process is based on the trimming of chemical patterns obtained by argon fluoride (ArF) immersion lithography. After the deposition and cross-link of the PS layer, positive-tone photoresist patterns are created on top of it by ArF immersion lithography. At this point, the pattern consisting of the photoresist lines is trimmed, transferred into the cross-linked PS layer, and stripped with a strong solvent. Subsequently, a neutral layer is deposited and grafted on the substrate. In this way, the chemical patterns consisting of PS guidelines and a grafted neutral layer background are formed. Finally, the BCP thin film is deposited on the chemical pattern and thermally treated in order to achieve the phase separation in perpendicularly oriented lamellae [15].

Regarding the SMART process, a photo-resist layer is deposited on top of the PS-*r*-PMMA cross-linked layer. On top of this photo-resist layer, a lithography process is performed in order to transfer the pattern through the PS-*r*-PMMA layer by means of dry etching. After the stripping of the photoresist, a pattern consisting of zones with a PS-*r*-PMMA layer and zones where the substrate is exposed are formed. Subsequently, selective grafting of the functionalized polymer is performed in order to create the guides affine to one of the two blocks of the PS-*b*-PMMA BCP in order to achieve the DSA of the lamellae when the PS-*b*-PMMA layer is deposited on it [16].

Finally, for what concerns the COOL process, the guide pattern consists of line/space structures obtained by ArF immersion lithography of a photoresist. Subsequently, the resist pattern is etched in order to slim the guides and slightly etch the substrate. This etching step modifies the surface energy of the resist guide, making it more affine to the PMMA block of the BCP. In this way, the resist guide acts as a directional guide for the subsequent DSA of the lamellae BCP. At this point, the selective grafting of functionalized PS-*r*-PMMA is performed. The grafting process takes place exclusively on the substrate, leaving the properties of the guides unaltered. In the end, the BCP layer is deposited and baked on the top of the chemical pattern. In this case, the resist guides act as directional guidelines for the DSA [17].

The principal limitation of these methods in the integration of the DSA BCPs chemo-epitaxy process in 300 mm fab is related to the use of polymeric pining materials to fabricate the guideline for the DSA. This can limit the control of the surface free energy and the final critical dimension of the guidelines [17,18].

This work reports a novel, simple, and resist-free chemo-epitaxy process permitting the DSA of lamella forming BCPs on the 300 mm wafer. This process is called the “Trim-Ox approach”. Here, conventional lithography is used to manufacture topographical tetraethyl orthosilicate (TEOS) line/space patterns. The TEOS lines were exploited to create the PS guidelines that permit achieving the DSA. The critical dimension (CD) of the obtained TEOS lines can be easily trimmed by means of wet etching: it allows a good control of the CD that permits finely tuning the guideline and the background dimensions. The evolution of key metrics—period (L), LER, LWR, and Orientational Parameter—are evaluated as a function of the TEOS pitch size. The possibility of obtaining a sub-20 nm critical dimension guideline allows the integration of BCPs having a period below 20 nm.

## 2. Experimental

### 2.1. Materials

The BCP studied was a lamella-forming poly(styrene-block-methylmethacrylate) (PS-*b*-PMMA) formulated in propylene glycol methyl ether acetate (PGMEA) that was synthesized by ARKEMA under the trend name Nanostrength^®^ EO. The BCP has an intrinsic period of 32 nm [18], and it is referred to as L32 in this work. The guideline was a functionalized PS that can graft the substrate to form a compact brush layer. Four different polystyrene-random-methylmethacrylate (PS-*r*-PMMA) backgrounds were used:

- Cross-linked PS-*r*-PMMA (NLa) layer
- Functionalized PS-*r*-PMMA (NLb) with high molar mass
- Functionalized PS-*r*-PMMA (NLc) with low molar mass
- Functionalized PS-*r*-PMMA (NLd) with low molar mass (comparable to NLc) and different fractions of PS with respect to NLc.

All polymers were synthetized by the industrials partners, Arkema and Brewer Science, and used as received.

For the lithography stacks, silicon anti-reflective coatings (SiARC), spin-on-carbon (SOC), and tetraethyl orthosilicate (TEOS) layers were employed. Additionally, a lithography step was realized with a chemically amplified negative resist developed in the negative tone using TMAH.

All process steps presented thereafter were performed on the LETI’s 300 mm pilot line. More precisely, coating, annealing, and wet treatment were carried out on the DSA-dedicated SCREEN RF3 and Sokudo Duo tracks.

The etching process with hydrofluoric (HF) acid 1% was performed by using the RAIDER 4B tool for 300 mm wafer.

### 2.2. Characterization

The top-view critical dimension scanning electron microscopy (CD-SEM) images were obtained using a HCG4000 CD-SEM from Hitachi with an accelerating voltage of 500 V. The fraction of the perpendicularly oriented lamellae and intrinsic period (L) of the BCP were obtained by means of the analysis of the CD-SEM images with ImageJ software. LER, LWR, and Herman’s orientational parameter (P) were obtained by means of ADAblock software [19].

The film thicknesses measurement was performed by ellipsometry methods with an Atlas XP+ tool from Nanometrics.

## 3. Results

**Figure 1:**
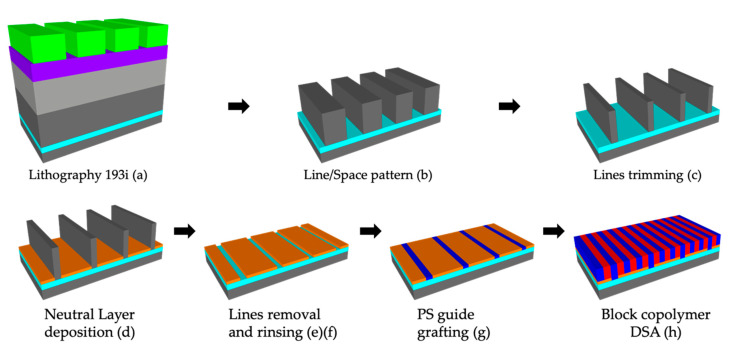
Schematic representation of trim-ox process flow. (**a**) Lithography step where the stack consists of, starting from the bottom: tetraethyl orthosilicate (TEOS) (140 nm)/titanium nitride (TiN) (30 nm)/TEOS (140 nm)/spin-on-carbon (SOC) (140 nm)/silicon anti-reflective coatings (SiARC) (35 nm)/lithography resist (100 nm); (**b**) line/space pattern (pitch 97.5 ± 1 nm to 200 ± 1 nm with a step of 2.5 nm); (**c**) lines trimming with hydrofluoric (HF) 1% (critical dimension (CD) = 15 ± 1 nm); (**d**) neutral layer spin coating (**e**,**f**) lines removal with HF 1% and rinsing with propylene glycol methyl ether acetate (PGMEA); (**g**) selective grafting of polystyrene (PS); (**h**) directed self-assembly (DSA) of BCP L32.

Figure 1 summarized the process steps for the DSA of the lamellar PS-*b*-PMMA on patterned 300 mm wafers. A stack consisting of tetraethyl orthosilicate (TEOS)/spin-on carbon (SOC)/silicon anti-reflective coating (SiARC)/lithography resist is deposited on 30 nm thick titanium nitride (TiN) substrate (Figure 1a). Then, a 140 nm TEOS line/space pattern (Figure 1b) was obtained by means of 193i lithography on the TiN substrate. The pitch of the TEOS lines varies from 97.5 ± 1 nm to 200 ± 1 nm with a step of 2.5 nm, while the CD of the lines can be easily optimized by means of HF etching at 1% (Figure 1c). At this point, the neutral layer PS-*r*-PMMA is deposited by means of the spin-coating method (Figure 1d), TEOS lines are removed by HF etching at 1% (Figure 1e), and PGMEA rinsing is performed. Subsequently, functionalized PS is selectively grafted to form the guideline and rinsed with PGMEA (Figure 1f). Finally, the BCP L32 is deposited by spin-coating and annealed in order to obtain the self-assembly (Figure 1h).

**Figure 2:**
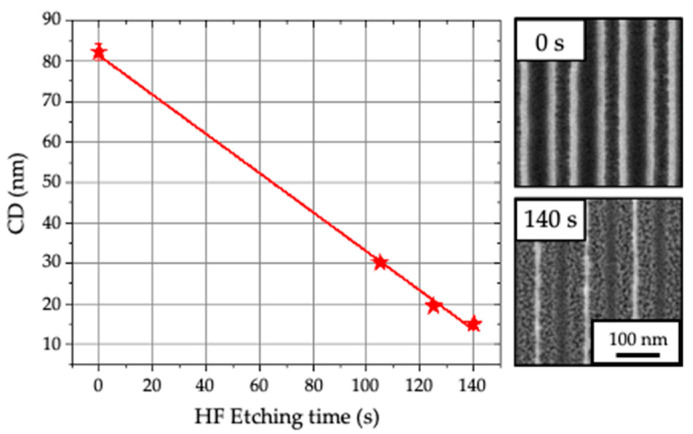
Lateral etching rate of TEOS lines with HF 1% and representative CD-SEM plan-view images of TEOS lines before etching (0 s) and after 140 s.

Figure 2 shows the lateral etching rate evolution of TEOS lines in line/space pattern having a pitch of 120 ± 1 nm measured from top-view CD-SEM images where the CD of the TEOS lines pass progressively from 82 ± 2 nm to 15 ± 1 nm as a function of the HF etching time. This process step is referred to the lines trimming sketched in Figure 1c. The final etching rate is 0.25 nm/s (15 nm/min) on 300 mm wafer, which is three times faster than standard silicon dioxide (SiO_2_) [20].

The CD of the TEOS lines defines the CD of the PS guiding lines. In order to obtain a long-range ordering of the BCP L32, the CD of the lines must be optimized at 15 nm, which represents the natural half period (L_0_/2) of one BCP lamella [9]. For this reason, the HF etching time was fixed at 140 s.

**Figure 3:**
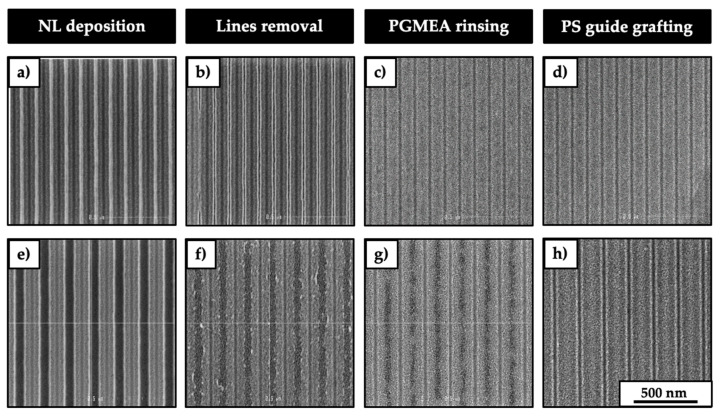
Representative plan view CD-SEM images of the steps: NLa deposition (**a**), NLb, NLc, NLd (**e**), lines removal with HF (**b**,**f**), PGMEA rinsing (**c**,**g**), and PS guide grafting (**d**,**h**).

At this point, the 300 mm wafers are spin coated with the selected neutral layers by using the SCREEN RF3 track and then annealed by using the SCREEN Sokudo DUO track. Figure 3 shows a representative plan view CD-SEM images of steps d, e, f, and g sketched in Figure 1.

The thicknesses of NLa, NLb, NLc, and NLd were adjusted optimizing the spin coating parameters. For NLa, the thermal treatment achieves the cross-linking of the polymeric chains inside the polymeric film while for NLb, NLc, and NLd, it promotes the grafting of the chains on the substrate. These experimental parameters are summarized in Table 1.

Subsequently, the removal of the TEOS lines is mandatory to graft the PS and form the guidelines. In this context, the etching with HF 1% was optimized to 180 s to completely remove the TEOS lines (Figure S1, supplementary information). Here, it is possible to notice that in the case of NLa (Figure 3b), the guide left by the TEOS lines having the pitch of 120 nm is clearly visible and the edges are well defined. On the other hand, the guide left by the TEOS lines with the brush NLb, NLc, and NLd are not clearly visible in the pattern having a pitch of 120 nm. For this reason, the CD-SEM images are representative for the patterns having the pitch of 180 nm (Figure 3f). The next step consists in the PGMEA rinsing of the 300 mm wafer in order to remove the particles created during the etching of the TEOS lines and to remove the ungrafted polymer chains in the case of NL brushes. Figure 3c,g shows the effect of the PGMEA rinsing on the neutral layer. In the case of NLa (Figure 3c), the rinsing causes the shrinkage of the guides, making problematic the analysis with CD-SEM. For the brushes NLb, NLc, and NLd (Figure 3g) the guide is still visible, but the edges of the guides are not well defined. Finally, the grafting of the PS (Figure 3d,h) permits creating the guide necessary to have a chemo-epitaxy pattern for the subsequent DSA of the L32 BCP. The concentration of the PS solution and the spin-coating parameters were adjusted in order to obtain a thickness of 15 nm.

After the thermal treatment at 200 °C for 75 s and the subsequent PGMEA rinsing, the resulting PS-grafted layer thickness was around 4 nm. In both cases, the contrast is not enough to measure the CD of the PS guidelines, but the CD of these guidelines is defined by the CD of the TEOS lines previously measured by CD-SEM.

The last step of the trim-ox approach regards the deposition of the BCP L32 on the chemical pattern by using the Sokudo DUO track (Figure 1h). The concentration of the solution and the spin coating parameters were adjusted to obtain a BCP film thickness of 33 nm. Then, the wafers were thermally annealed at 240 °C for 900 s to achieve the self-assembly of the L32 film in perpendicularly oriented lamellae.

**Figure 4:**
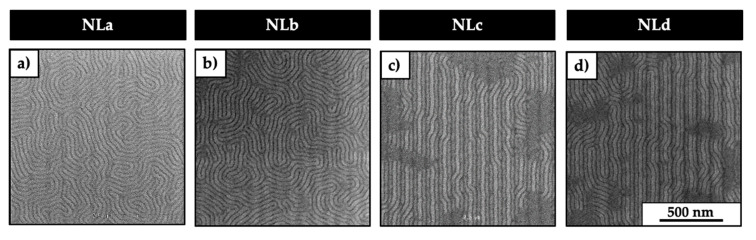
Representative top-view CD-SEM images of the self-assembled films when using NLa (**a**), NLb (**b**), NLc (**c**), and NLd (**d**) as neutral layers.

Figure 4 represents the top-view CD-SEM images of the self-assembled films for the four neutral layer employed (NLa, NLb, NLc, and NLd) for a TEOS lines pitch of 97.5 nm.

**Figure 5:**
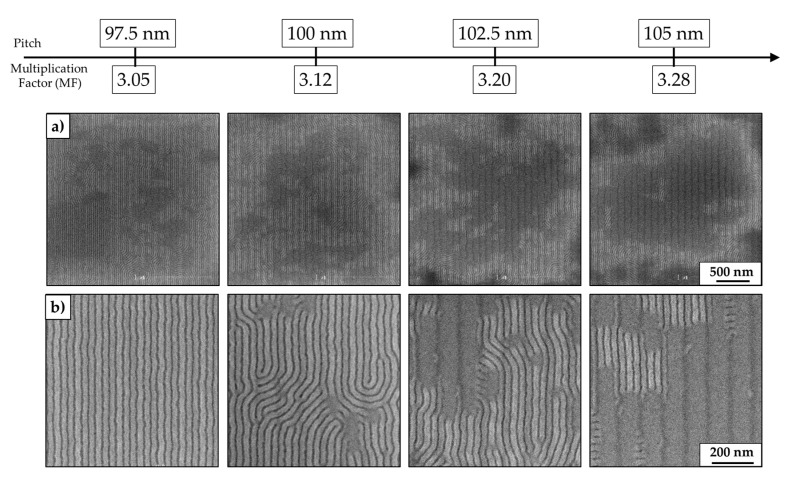
Representative CD-SEM images at low (**a**) et high (**b**) magnification, depicting the influence of the pitch dimension on the morphology of the BCP L32 for fixed CD ≈15 nm of TEOS lines. The multiplication factor (MF) is the result of Pitch/L_0_, where L_0_ = 32 nm is the period of L32 on a flat substrate.

Here, it is possible to notice that in the case of NLa and NLb, as reported in Figure 4a,b, respectively, the chemical pattern does not guide the lamellae of the BCP, and fingerprint morphology was observed. On the other hand, when the neutral layers employed are the NLc and NLd, the perpendicular lamellae starts to be guided by the chemical pattern, and in these cases, the DSA takes place. This is probably due the low molar masses of NLc and NLd, which leads to the formation of a compact brush layer, and it avoids the PS molecules to penetrate inside the brush neutral layer. By the analysis of the CD-SEM images, the fraction of perpendicular lamellae for NLc and NLd was calculated to be about 77 ± 4% and 88.5 ± 4.4%, respectively. Considering the latest results, the NLd was selected as a reference neutral layer, and the influence of pitch dimensions for fixed CD = 15 nm of the TEOS lines were investigated, as shown in Figure 5.

Here, the dimension of the pitch varies from 97.5 ± 1 nm to 105 ± 1 nm corresponding to a MF from 3.05 to 3.28. The dimension of the pitch influences the perpendicular orientation with respect to the substrate (Figure 5a) and DSA (Figure 5b) of the BCP L32 lamellae. In the first case, it is possible to notice the presence of dark zones on the patterns corresponding to the parallel alignment of the BCP L32 lamellae with respect to the substrate. The area of these zones augments progressively passing from a pitch of 97.5 ± 1 to 105 ± 1 nm. In the latest, the alignment of the lamellae perpendicularly oriented passes from well-aligned (pitch 97.5 nm) to not aligned lamellae (pitch 105 nm) with respect to the chemical pattern pitch.

**Figure 6:**
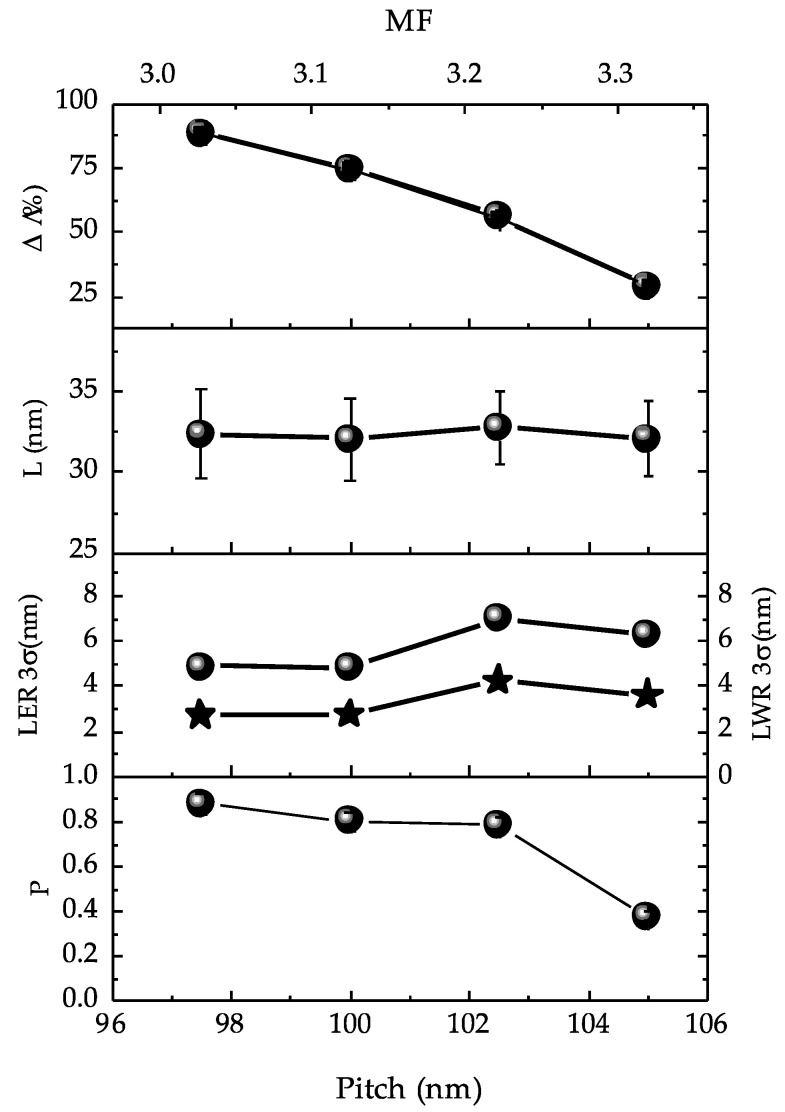
Evolution of Δ (%), period L, line edge roughness (LER) (stars), line width roughness (LWR) (spheres), and Herman’s orientational (P) parameter as a function of the TEOS pitch size and multiplication factor (MF) in L32 BCP thin films.

Figure 6 reports the fractions of the perpendicularly oriented lamellae with respect to the substrate Δ (%) and the evolution of key metrics: period (L), Lines edge roughness (LER), Lines width roughness (LWR), and Herman’s Orientational Parameter (P) as a function of TEOS lines pitch. These data are the results of the analysis of the CD-SEM images reported in Figure 5. The first graph from the top of the stack indicates the fraction of perpendicularly oriented lamellae (% Δ) as a function of the pitch. In the case, the fraction of lamellae perpendicularly oriented with respect to the substrate progressively decreases from 88.5 ± 4.4% to 28.9 ± 1.4% passing from a pitch of 97.5 ± 1 to 105 ± 1 nm of the TEOS lines.

The second graph of the stack in Figure 6 reports the evolution of L as a function of the pitch. Here, it is possible to notice that L remain constant around values of ≈32 ± 2 nm independently of the pitch of the TEOS lines employed.

The third graph of each stack reports the LER (3σ) and LWR (3σ) evolution as a function of the pitch considering the systems with lamellae perpendicularly oriented with respect to the substrate. LER and LWR represent the deviation from a straight-line edge and the deviation from a uniform line width, respectively [19,21]. In this case, the LER and LWR values fluctuate in the range of LER ≈2.7–4.2 nm and LWR ≈4.8–7 nm. In particular, for the pitch of 97.5 ± 1 and 100 ± 1 nm, respectively, these values remain constant with nominal values of LER ≈2.7 nm and LWR ≈4.8 nm. For a pitch of 102.5 nm, the LER and LWR tend to increase to values of ≈4.2 nm and ≈7 nm, respectively. Finally, for a pitch of 105 nm, the LER and LWR decrease, reaching values of 3.6 nm and 6.2 nm, respectively. This morphological evolution is characteristic of non-commensurability between the chemical pattern and the period of the BCP [9].

The fourth graph of each stack reports the Herman’s Orientational Parameter (P) evolution as a function of TEOS pitch. The *P* parameters [22,23] gives a measure of the uniformity of the lamellae within an image. In this case, the more the *P* parameter tends to 1, the more the lamellae are oriented vertically following the chemical patterns created on the substrate [19,23].

The *P* values of the perpendicular lamellae decrease with the TEOS pitch size passing from *P* = 0.879 ± 0.044 for a pitch of 97.5 ± 1 nm to *P* = 0.378 ± 0.019 for a pitch of 105 ± 1 nm. These values of *P* indicate a progressive loss of the DSA with the increasing of the pitch size because the *P* parameter tends to move away from the values of *P* = 1, which indicates the perfect ordering of the lamellae nanostructures.

## 4. Discussions

The DSA of the lamella forming PS-*b*-PMMA is affected by the nature of the neutral layers employed. In the case of cross-linkable (NLa) and highest molar mass brush (NLb) neutral layers, the chemical contrast between the PS guidelines and the NL background is not enough to drive the DSA of the BCP. In the first case, this is due to the shrinkage of the NLa after the PGMEA rinsing that limits the possibility of having the PS guideline (Figure 3b,c). In the second case, this is probably due to the higher molar mass of the brush neutral layer compared to the molar mass of the PS that forms the guideline. This is probably because the interface between the polymeric chains of the PS guideline and the NL brushes is not clear-cut because of an interpenetration between the polymeric chains of NL and PS that does not permit obtaining the DSA of the perpendicular lamellae. In fact, for NLc and NLd, the neutral layers have molar mass comparable to the one of the polymer that forms the PS guideline. In this case, the chemical pattern created guides the perpendicular-oriented lamellae, achieving the DSA. As previously reported in the literature, experimental [24,25] and computational [26] studies confirmed that the interpenetration region between systems consisting of polymeric films deposited onto grafted brush layers increases with the molar mass of the brush layer by fixing the molar mass of the polymer film. Although these previous studies investigated the interpenetration between layers and not the lateral interpenetration, they can be useful to give a plausible explanation to the results obtained in the present work.

The overall picture of the reported data demonstrate that the PS guideline of the chemo-epitaxial pattern can be obtained exploiting the trimming and the following etching of TEOS L/S patterns without the use of polymeric pinning materials, which is commonly exploited in the existing DSA methods.

In this context, considering the SMART process, the photoresist is deposited, etched, and stripped on top of the cross-linked PS-*r*-PMMA layer to form the guidelines. The critical dimension (CD) of the guidelines is defined by the photolithographic methods. Therefore, this introduces limitations in terms of the CD range that is accessible. Moreover, the etching process to transfer the pattern into the neutral layer and the stripping process to remove the photoresist could cause modifications in the surface properties of the resulting neutral patterns. In a similar way, the LiNe process fabricated the guideline, but in this case, the limits of the CD were overcome by means of the O_2_ plasma trimming process of the resist to reduce its CD. On the other side, the trimming process introduces changing in the affinity with respect to one block of the BCP. The COOL process is very similar to the LiNe process, but the guideline is represented by the resist that is slimmed by means of the etching process. Thus, there no need to strip the resist during the process. Nevertheless, the affinity of the resist guideline with one of the BCP blocks is guaranteed by the etching process, and these can introduce limitations regarding its univocal affinity with one of the BCP blocks.

According to the International Technology Roadmap for Semiconductor (ITRS) [27] and the International Roadmap for Devices and Systems (IRDS) [28], the LER and LWR of nanostructures that have dimensions below 15 nm are requested to be lower than 0.9 and 1.2 nm, respectively, because higher values could afflict the performance of the final microelectronics devices. In particular, high values are deleterious for circuit elements. For transistor gate structures, the roughness causes significant variations in the off-current, as well as affecting threshold voltages. For nanometer-scale interconnects, the roughness increases both resistance and capacitance [21,29,30,31]. Table 2 reports the LER and LWR literature values for lamellae PS-*b*-PMMA nanostructures having a period L comparable to the one reported in the following work by a different DSA approach. Although the experimental LER and LWR values are almost 2–3 times higher than the limit values imposed by ITRS and IRDS, it is possible to notice that the LER and LWR values obtained with the Trim-Ox approach presented in this work are in line with the literature data of the most studied approach. Consequently, the Trim-Ox approach could be considered as a competitor method for the integration of the DSA in the existing conventional photolithography.

## 5. Conclusions

The Trim-Ox approach was implemented in order to obtain the DSA of BCP L32 having an intrinsic period of 32 nm. It has demonstrated the possibility to finely tune the CD dimension of the TEOS line with a simple etching in HF 1%, reaching the CD = 15 nm. Moreover, it has been demonstrated that the integration of chemical patterns composed of grafted NLd and PS guide on a 300 mm wafer can be achieved exploiting the TEOS lines. The process developed is fully compatible with 300 mm clean-room facilities, and the performance in terms of roughness can be considered at the same level to the state-of-the-art methods reported in the literature.

## 6. Patents

Patent related to this work is pending, application number FR1911542.

## Figures and Tables

**Table 1:** Experimental parameters used for the neutral layer deposition.

Neutral Layer	Thickness Deposited (nm) (±0.5)	Thickness after Rinse (nm) (±0.5)	Thermal Treatment
NLa	7	7	250 °C/300 s
NLb	30	7	200 °C/75 s
NLc	15	4	200 °C/75 s
NLd	15	4	200 °C/75 s

**Table 2:** LER and LWR values for various approaches that include LiNe, SMART, COOL, and the Trim-Ox approach presented in the following work.

DSA Approach	LER (3σ) (nm)	LWR (3σ) (nm)
LiNe [32,33,34,35]	2.49–3.12	2.45–4.0
SMART [36,37]	3.5–4.0	2.9–5.0
COOL [17,38]	3.5–4.5	4.0–6.0
Trim-Ox	2.7–4.2	4.8–7

## Supplementary Materials

The following are available online at https://www.mdpi.com/2079-4991/10/12/2443/s1, Figure S1: Evolution of the TEOS lines etching as function of the HF etching time.
