# Supplementary material for: Resist-Free Directed Self-Assembly Chemo-Epitaxy Approach for Line/Space Patterning
Source: Nanomaterials (Basel). 2020 Dec 7;10(12):2443. doi: 10.3390/nano10122443 (PMC7762273; doi:10.3390/nano10122443)
Supplement: Supplementary file 1 [file nanomaterials-10-02443-s001.pdf]

nanomaterials

Article

Resist-Free Directed Self-Assembly Chemo-Epitaxy Approach for Line/Space Patterning

Tommaso Jacopo Giammaria [1,*], Ahmed Gharbi [1], Anne Paquet [1], Paul Nealey [2] and Raluca Tiron [1]

[1] Université Grenoble Alpes, CEA, Leti, F-38000 Grenoble, France; ahmed.gharbi@cea.fr (A.G.); anne.paquet74@gmail.com (A.P.); raluca.tiron@cea.fr (R.T.)
[2] Institute for Molecular Engineering, University of Chicago, 5747 South Ellis Avenue, Chicago, IL 60637, USA; nealey@uchicago.edu
* Correspondence: tommaso.giammaria@cea.fr

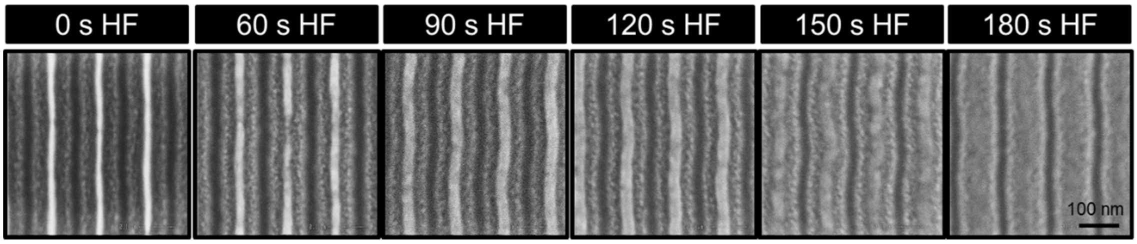

Figure S1. Evolution of the TEOS lines etching as function of the HF etching time.

© 2020 by the authors. Submitted for possible open access publication under the terms and conditions of the Creative Commons Attribution (CC BY) license (http://creativecommons.org/licenses/by/4.0/).
